# Granulocyte and astrocyte markers distinguish MOG-antibody disease and neuromyelitis optica from multiple sclerosis

**DOI:** 10.1093/brain/awaf345

**Published:** 2025-09-24

**Authors:** Roberto Furlan, Sabine Schaedelin, Jette Lautrup Frederiksen, Mitsuru Watanabe, Noriko Isobe, Fredrik Piehl, Katharina Fink, Ellen Iacobaeus, Björn Evertsson, Mohsen Khademi, Matteo Gastaldi, Giacomo Greco, Sara Mariotto, Sara Carta, Alessia Di Sapio, Cecilia Irene Bava, Lucia Giorgi, Pascal Benkert, Aleksandra Maleska Maceski, Johanna Oechtering, Eline Willemse, Anne-Katrin Pröbstel, Roxanne Pretzsch, Annamaria Finardi, Alessandra Mandelli, Daniel C Anthony, Jens Kuhle, David Leppert

**Affiliations:** Clinical Neuroimmunology Unit, Institute of Experimental Neurology, Division of Neuroscience, IRCCS Ospedale San Raffaele, and Vita e Salute San Raffaele University, Milan 20132, Italy; Department of Clinical Research, University Hospital Basel, University of Basel, Basel 4031, Switzerland; Department of Neurology at Rigshospitalet and Institute of Clinical Medicine, University of Copenhagen, Copenhagen 2100, Denmark; Department of Neurology, Neurological Institute, Graduate School of Medical Sciences, Kyushu University, Fukuoka 812-8582, Japan; Department of Neurology, Neurological Institute, Graduate School of Medical Sciences, Kyushu University, Fukuoka 812-8582, Japan; Department of Clinical Neuroscience, Karolinska Institutet and Department of Neurology, Karolinska University Hospital, Stockholm SE-171 76, Sweden; Department of Clinical Neuroscience, Karolinska Institutet and Department of Neurology, Karolinska University Hospital, Stockholm SE-171 76, Sweden; Department of Clinical Neuroscience, Karolinska Institutet and Department of Neurology, Karolinska University Hospital, Stockholm SE-171 76, Sweden; Department of Clinical Neuroscience, Karolinska Institutet and Department of Neurology, Karolinska University Hospital, Stockholm SE-171 76, Sweden; Department of Clinical Neuroscience, Karolinska Institutet and Department of Neurology, Karolinska University Hospital, Stockholm SE-171 76, Sweden; Multiple Sclerosis Center, IRCCS Mondino Foundation, and Department of Brain and Behavioural Sciences, University of Pavia, Pavia 27100, Italy; Multiple Sclerosis Center, IRCCS Mondino Foundation, and Department of Brain and Behavioural Sciences, University of Pavia, Pavia 27100, Italy; Neurology Unit, Department of Neurosciences, Biomedicine and Movement Sciences, University of Verona, Verona 37134, Italy; Neurology Unit, Department of Neurosciences, Biomedicine and Movement Sciences, University of Verona, Verona 37134, Italy; Department of Neurology, Regional Referral MS Center and CRESM BioBank, San Luigi Gonzaga University Hospital, Orbassano 10043, Italy; Department of Neurology, Regional Referral MS Center and CRESM BioBank, San Luigi Gonzaga University Hospital, Orbassano 10043, Italy; Department of Neurology, Regional Referral MS Center and CRESM BioBank, San Luigi Gonzaga University Hospital, Orbassano 10043, Italy; Department of Clinical Research, University Hospital Basel, University of Basel, Basel 4031, Switzerland; Multiple Sclerosis Centre and Research Center for Clinical Neuroimmunology and Neuroscience (RC2NB), Departments of Biomedicine and Clinical Research, and Department of Neurology, University Hospital and University of Basel, Basel 4031, Switzerland; Multiple Sclerosis Centre and Research Center for Clinical Neuroimmunology and Neuroscience (RC2NB), Departments of Biomedicine and Clinical Research, and Department of Neurology, University Hospital and University of Basel, Basel 4031, Switzerland; Multiple Sclerosis Centre and Research Center for Clinical Neuroimmunology and Neuroscience (RC2NB), Departments of Biomedicine and Clinical Research, and Department of Neurology, University Hospital and University of Basel, Basel 4031, Switzerland; Center of Neurology, Department of Neuroimmunology, University Hospital and University Bonn, Bonn 53127, Germany; Multiple Sclerosis Centre and Research Center for Clinical Neuroimmunology and Neuroscience (RC2NB), Departments of Biomedicine and Clinical Research, and Department of Neurology, University Hospital and University of Basel, Basel 4031, Switzerland; Clinical Neuroimmunology Unit, Institute of Experimental Neurology, Division of Neuroscience, IRCCS Ospedale San Raffaele, and Vita e Salute San Raffaele University, Milan 20132, Italy; Clinical Neuroimmunology Unit, Institute of Experimental Neurology, Division of Neuroscience, IRCCS Ospedale San Raffaele, and Vita e Salute San Raffaele University, Milan 20132, Italy; Department of Pharmacology, University of Oxford, Oxford OX1 3QT, UK; Multiple Sclerosis Centre and Research Center for Clinical Neuroimmunology and Neuroscience (RC2NB), Departments of Biomedicine and Clinical Research, and Department of Neurology, University Hospital and University of Basel, Basel 4031, Switzerland; Multiple Sclerosis Centre and Research Center for Clinical Neuroimmunology and Neuroscience (RC2NB), Departments of Biomedicine and Clinical Research, and Department of Neurology, University Hospital and University of Basel, Basel 4031, Switzerland

**Keywords:** CSF biomarkers, translational research, autoimmunity, complement activation

## Abstract

Granulocytes play a well-established role in the pathogenesis of brain tissue damage in neuromyelitis optica spectrum disorder (NMOSD). The release of granulocyte activation markers (GAM) into CSF has recently been shown to distinguish NMOSD from multiple sclerosis (MS) with high accuracy. However, their pathogenetic role in myelin oligodendrocyte glycoprotein antibody-associated disease (MOGAD) is less clear, and their usefulness for diagnostic differentiation is unknown.

This observational cohort study by eight tertiary centres in Europe and Japan included 244 CSF samples from patients with MOGAD (*n* = 71), NMOSD (*n* = 48), MS (*n* = 125) and control persons (*n* = 19). CSF levels of GAM [neutrophil elastase, myeloperoxidase, neutrophil gelatinase-associated lipocalin (NGAL), matrix metalloproteinase-8 and 9 (MMP-8, MMP-9)], astrocyte damage markers [ADM: glial fibrillary acidic protein (GFAP), S100B], and complement factors C5 and C5a were analysed by capillary ELISA (Ella™) or Luminex®. The primary outcome was the capacity of these markers to differentiate MOGAD, NMOSD and MS in the acute (≤21 days post-exacerbation) stage, and the correlation of GAM with C5 and C5a. Secondary analyses included the correlations of these markers with disability severity, measured by the Expanded Disability Status Scale (EDSS).

GAM (except for MMP-9), ADM and C5/C5a levels peaked at onset of disease exacerbation of MOGAD and NMOSD (regardless of aquaporin-4 antibody status), and were significantly higher than in MS. MMP-9 levels were continuously increased in MS over MOGAD and NMOSD, both in acute and subacute/chronic stages. C5 and C5a were equally increased over MS in acute stages of MOGAD and NMOSD. A logistic model and receiver operating characteristics analyses incorporating GAM and C5 displayed high discriminatory power between MOGAD/NMOSD versus MS [area under the curve (AUC) = 0.880], NMOSD versus MS (AUC = 0.837) and MOGAD versus MS (AUC = 0.925) in acute stages. Accordingly, increased ADM levels in NMOSD differentiated NMOSD from MS and MOGAD (AUC = 0.897 and 0.843, respectively). GAM levels correlated with EDSS scores in MOGAD and NMOSD, but not in MS, while those of ADM correlated with disability in NMOSD, but not in MOGAD and MS.

Determining CSF levels of GAM and C5/C5a, and of ADM provide a biology-driven approach to differentiate MOGAD, NMOSD and MS. Their measurement can be processed faster and with similar accuracy than with most autoantibody assays, enabling timely initiation of appropriate therapy in acute presentations. The correlation between GAM and C5/C5a levels with neurological impairment in MOGAD and NMOSD corroborates their role as effectors of neural damage, supporting the acute stage use of inhibitors of C5 activation.

## Teaser

Furlan *et al*. demonstrate that CSF profiles of granulocyte activation and astrocyte damage markers, and complement factor C5 differentiate neuromyelitis optica spectrum disorder (NMOSD), myelin oligodendrocyte glycoprotein antibody associated disease (MOGAD) and multiple sclerosis, independent of autoantibody tests.

**See Paul (https://doi.org/10.1093/brain/awag093) for a scientific commentary on this article.**

## Introduction

Myelin oligodendrocyte glycoprotein antibody-associated disease (MOGAD), neuromyelitis optica spectrum disorder (NMOSD) and multiple sclerosis (MS) share clinical and imaging characteristics in their acute stages, often making diagnosis challenging. This diagnostic uncertainty can lead to delays in initiating appropriate therapy or the administration of disease-modifying treatments for MS that may exacerbate NMOSD and MOGAD. Specifically, interferons, natalizumab, fingolimod and dimethyl fumarate have been shown to worsen outcomes in patients with these conditions.^1-3^ Conversely, an expedited start of therapy is key for the prevention of short- and long-term disability in acute exacerbations of NMOSD^4-6^ and MOGAD,^7^ yet there are currently no diagnostic biofluid markers established that meet clinical accuracy standards in these circumstances.

A key pathological feature that differentiates MOGAD and NMOSD from MS is the presence of granulocyte invasion and complement activation in brain lesions.^8-11^ However, despite this understanding, no diagnostic tools or targeted therapies for the acute disease stage have been developed based on these mechanisms. During inflammatory activation, granulocytes release pre-stored granules collectively referred to as granulocyte activation markers (GAM): neutrophil elastase (nEla), myeloperoxidase (MPO) (from primary granules), matrix metalloproteinase (MMP)-8 and neutrophil gelatinase-associated lipocalin (NGAL)/lipocalin-2 (from secondary granules) are cell-specific products of granulocytes in the context of neurological disease. In contrast, MMP-9 (from tertiary granules) is also produced and released by many other cells.^12^ We have recently shown that elevated levels of GAM in CSF of acute NMOSD allows the differentiation versus MS, irrespective of aquaporin-4 antibody (aAQP4) serostatus, with equal sensitivity and specificity as cell-based assays for aAQP4.^12^ Furthermore, GAM are the first biomarkers whose levels correlate with disability scores in acute NMOSD,^12^ mirroring results of their functional role for neural tissue damage in referring animal models,^13-16^ while little is known about their role in MOGAD.

In this case-control study we investigated CSF levels of GAM, complement factors C5 and C5a, neuronal [neurofilament light chain (NfL)] and astrocyte damage markers [ADM: glial fibrillary acidic protein (GFAP), S100B] as diagnostic biomarkers differentiating MOGAD versus NMOSD and MS, and how they relate to functional neurological impairment.

## Materials and methods

### Participants and samples

The diagnosis of MOGAD, NMOSD with or without aAQP4, and of MS was based on respective standard diagnostic criteria.^17-19^ Myelin oligodendrocyte glycoprotein antibodies (aMOG) and aAQP4 were measured with cell-based assays at each site. Acute disease exacerbation/relapse was defined according to 2017 McDonald criteria.^19^ Disability was assessed using the Expanded Disability Status Scale (EDSS).^20^

CSF samples were provided by Karolinska University Hospital (Stockholm, Sweden) (*n* = 85), San Luigi Gonzaga University Hospital Orbassano [Orbassano (Turin), Italy] (*n* = 36), Kyushu University Hospital (Fukuoka, Japan) (*n* = 46), Rigshospitalet University of Copenhagen (Denmark) (*n* = 36), Mondino Foundation (Pavia, Italy) (*n* = 20), University Hospital Basel (Switzerland) (*n* = 11) and the University of Verona (Italy) (*n* = 10). The study included a group of 19 ‘symptomatic controls’ (SC),^21^ in whom a structural neurological disease was excluded, based on normal clinical and MRI findings, normal CSF cell composition and protein content, and absence of signs of intrathecal immunoglobulin G (IgG) synthesis (further demographic and clinical data are provided in Supplementary Table 1). One patient in the MOGAD cohort contributed two samples; the lumbar punctures (LPs) were performed following independent disease exacerbations. Institutional review boards at the respective sites approved the study and written informed consent was obtained from all participants.

### Measurement of biomarkers

Standard CSF analyses were performed independently at each centre, while biomarker measurements were conducted centrally. Expression levels of GAM, ADM and NfL were determined by capillary ELISA (Ella™, Bio-Techne), except for nEla, and for C5/C5a, which were measured by Luminex® (Merck Life Science Srl). Assay operators were masked to sample identities until all analyses were completed. Each sample and calibrators were analysed in duplicate (Luminex) or triplicate (Ella). An exploratory analysis of biomarker levels across different collection sites indicated comparable values within each disease category and among SC (data not shown).

### Statistical analysis

CSF biomarker levels are presented as medians with interquartile ranges (IQR) by diagnostic group and compared using the Wilcoxon rank-sum test. To determine the capacity of distinguishing between MOGAD, NMOSD and MS without the potential confounding effect of therapy prior to LP, we repeated some analyses in treatment-naive patients. To investigate the temporal dynamics of biomarker concentrations, we used an individual linear model for each biomarker to describe the levels in each disease group within a 60-day period after acute disease exacerbation defined in days between the onset of acute disease exacerbation and LP. Biomarker levels were log-transformed and served as dependent variables. Diagnosis and time since disease exacerbation, as well as the interaction between these two variables, were used as independent variables. The interaction indicates whether the temporal dynamics differ between patients across disease groups. The correlation between biomarker levels and EDSS score was quantified using Spearman’s rank correlation coefficient. The diagnostic capacity of GAM and C5/C5a to differentiate MOGAD and NMOSD from MS in acute stages (≤21 days after onset of exacerbations) was determined by a logistic model where the disease type served as a dependent variable, and biomarkers, or their composites, as independent variables. The predictions from these models were assessed with receiver operating characteristic (ROC) curves. For each model, the area under the curve (AUC), as well as sensitivity and specificity, based on the optimal cut-off according to the Youden index, are presented. All analyses were carried out using the statistical software R (v.4.1.2, The R Foundation for Statistical Computing (https://www.r-project.org/foundation/). In accordance with the exploratory nature of these analyses, no correction for multiple testing was performed. Accordingly, *P*-values should not be interpreted as confirmatory but rather as a continuous measure of evidence against the corresponding null hypothesis. The significance level was set at *P* = 0.05.

## Results

### Demographics of patients and symptomatic controls

Table 1 presents the baseline characteristics of SC^21^ and patients with MOGAD, NMOSD and MS; patients were stratified into ‘acute’ (≤21 days) and ‘subacute/chronic’ (>21 days) stages, depending on the time between onset of acute clinical symptoms and LP. A total of 35.4% of NMOSD patients scored negative for aAQP4 at the time point of LP as per cell-based assays (one patient converted to aAQP4^+^ at a later relapse). Patients with NMOSD were older (*P* < 0.001) and had higher EDSS scores (*P*_NMOSDversusMOGAD_, _NMOSDversusMS_ < 0.001) than patients with MOGAD or MS. The percentage of NMOSD patients on therapy with steroids or immunomodulators before LP (66.7%; one patient had received stem cell transplantation), was more than 2-fold higher than in those with MOGAD (29.6%) and MS (18.4%) (*P*_both_ < 0.001), but was not different between MOGAD and MS (*P* = 0.074). Between disease groups, the median number of days between disease exacerbation and LP in acute and subacute/chronic stages were similar, respectively (Table 1 and Supplementary Fig. 1).

**Table 1:** Demographic and clinical characteristics of patient groups and symptomatic controls

	MOGAD	NMOSD	MS	Symptomatic controls^a^
Patients, *N*^b^	70	48	125	19
Female, *n* (%)	41 (57.7)	40 (83.3)	85 (68.0)	15 (78.9)
Samples, *N*; acute: subacute/chronic^b^	71 (40/31)	48 (31/17)	125 (45/80)	19
Age at CSF sampling, years, median [IQR]^c^	36.0 [28.4, 46.5]	48.5 [36.8, 59.0]	36.0 [28.0, 48.0]	32.0 [24.5, 36.0]
Disease duration at LP, years, median [IQR]^c^	0.1 [0.0, 2.3]	1.3 [0.4, 7.9]	1.5 [0.3, 9.1]	−
Interval between clinical attack and LP, days^d^				
Acute, median [IQR]	9 [4.75, 16.0]	9 [4.5, 13.5]	10 [6.0, 16.0]	−
Subacute/chronic, median [IQR]	32 [31.0, 62.5]	57 [34.0, 124.0]	77 [34.0, 208.0]	−
EDSS score at LP, median [IQR]^c^	3.0 [2.0, 4.0]	3.5 [2.5, 6.5]	2.0 [1.0, 3.0]	−
Autoantibody status, *n* (%)				
aAQP4^−^/aAQP4^+^	−	17^e^ (35.4)/31 (64.6)	−	−
aMOG	71 (100)	0^e^ (0/17)	−	−
Patients treated prior to LP, *n* (%)^f^	21 (29.6)	32 (66.7)	23 (18.4)	−
Immunomodulators	3 (4.2)	11 (22.9)	20 (16.0)	−
Corticosteroids	18 (25.4)	21 (43.8)	3 (2.4)	−

aAQP4 = aquaporin-4 antibody; aMOG = myelin oligodendrocyte glycoprotein antibody; EDSS = expanded disability status scale; IQR = interquartile range; LP = lumbar puncture; MOGAD = myelin oligodendrocyte glycoprotein antibody-associated disease; MS = multiple sclerosis; NMOSD = neuromyelitis optica spectrum disorder; SC = symptomatic controls.

^a^Symptomatic controls as defined by Teunissen *et al*.^21^

^b^One patient contributed two samples from independent exacerbations.

^c^Age differences between the three diseases were significant: NMOSD versus MOGAD and NMOSD versus MS (pairwise comparison, all *P* < 0.001). Similarly, differences in EDSS scores were significant (*P*_NMOSDversusMOGAD_ = 0.005, *P*_NMOSDversusMS_ < 0.001).

^d^The intervals between symptom onset and LP were comparable between acute phase (all three diseases) and subacute/chronic phase for NMOSD versus MOGAD, and MS versus NMOSD, while the difference for MS versus MOGAD was significant (*P* = 0.021) (Supplementary Fig. 1).

^e^All 17 aAQP4^−^ NMOSD patients scored negative for aMOG.

^f^The number of patients under treatment at LP was significantly higher for NMOSD versus MOGAD (*P* = 0.002) and NMOSD versus MS (*P* < 0.001), while it was similar between MOGAD versus MS (*P* = 0.669). Acute therapy with corticosteroids was not performed > 1 day prior to LP.

### Biomarker expression profiles in MOGAD, NMOSD, MS and SC

Statistical data of comparisons between SC and disease groups in acute and subacute/chronic stages are provided in Supplementary Table 2.

#### GAM levels: nEla, MPO, MMP-8, NGAL and MMP-9

**Figure 1:**
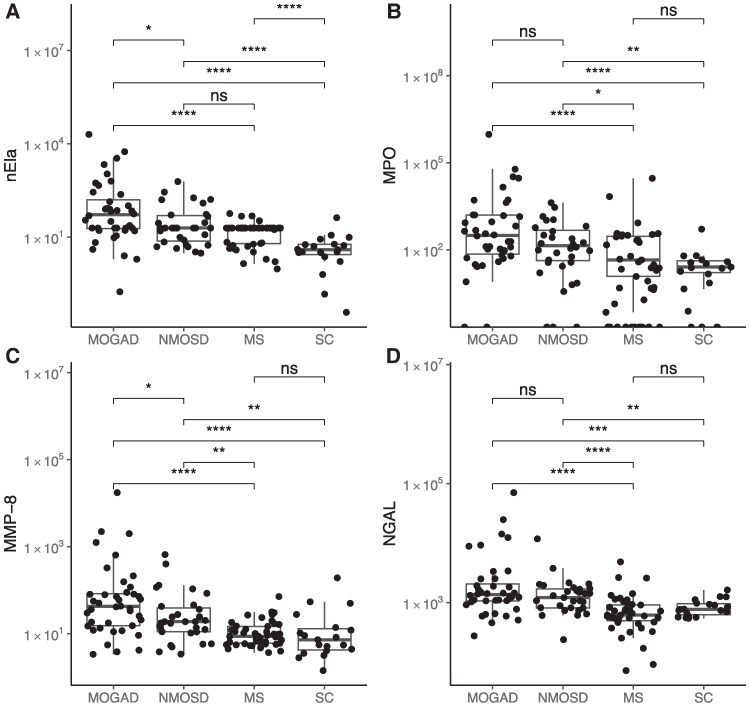
**CSF granulocyte activation markers (GAM) in acute myelin oligodendrocyte glycoprotein antibody-associated disease (MOGAD), neuromyelitis optica spectrum disorder (NMOSD), multiple sclerosis (MS) and symptomatic controls (SC)**. Box plots showing CSF concentrations of neutrophil elastase (nEla; **A**), myeloperoxidase (MPO; **B**), matrix metalloproteinase-8 (MMP-8; **C**) and neutrophil gelatinase-associated lipocalin (NGAL; **D**) across patient groups. Data are presented on a double logarithmic scale (*y*-axis; concentrations in pg/ml). Statistical comparisons were performed using the Wilcoxon rank-sum test; significance thresholds are denoted as: ns (not significant), *P* > 0.05; *P ≤ 0.05; **P ≤ 0.01; ***P ≤ 0.001; ****P ≤ 0.0001.

In acute stages, levels of nEla, MPO, MMP-8 and NGAL were increased in both MOGAD and NMOSD versus MS, and versus SC, except for nEla in NMOSD versus MS (*P* = 0.153); all GAM markers had median values significantly (nEla, MMP-8) or numerically (MPO, NGAL) higher in MOGAD versus NMOSD (Fig. 1). In contrast, in the subacute/chronic stage these markers had similar levels across diseases, and versus SC, except for nEla being increased in ascending order in NMOSD (*P* = 0.017), MS (*P* < 0.001) and MOGAD (*P* = 0.005) versus SC.

**Figure 2:**
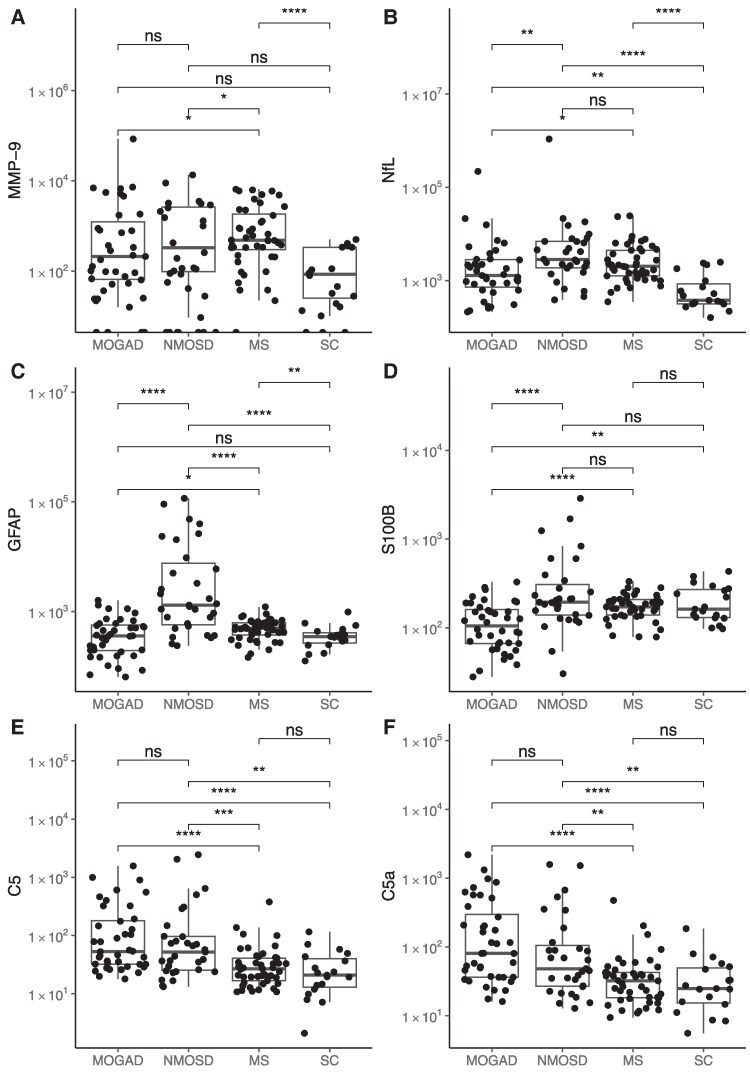
**CSF levels of MMP-9, tissue damage markers and complement factors in acute myelin oligodendrocyte glycoprotein antibody-associated disease (MOGAD), neuromyelitis optica spectrum disorder (NMOSD), multiple sclerosis (MS) and symptomatic controls (SC)**. Box plots showing CSF concentrations of matrix metalloproteinase-9 (MMP-9; **A**), neurofilament light chain (NfL; **B**), glial fibrillary acidic protein (GFAP; **C**), S100 calcium-binding protein B (S100B; **D**), complement factor C5 (**E**) and complement activation product C5a (**F**) across patient groups. Data are presented on a double logarithmic scale (*y*-axis; concentrations in pg/ml, except for C5 in ng/ml). Statistical comparisons were performed using the Wilcoxon rank-sum test; significance thresholds are denoted as: ns (not significant), *P* > 0.05; *P ≤ 0.05; **P ≤ 0.01; ***P ≤ 0.001; ****P ≤ 0.0001.

MMP-9 levels were increased in the acute stage of MS versus MOGAD, NMOSD and SC. Compared with acute stage MS, where levels were 10-fold higher than in SC, there was only a numerical increase of MMP-9 in MOGAD and NMOSD versus SC (Fig. 2). In the subacute/chronic stage, MMP-9 levels were increased in MS versus MOGAD (*P* = 0.005) and SC (*P* < 0.001), but not versus NMOSD.

#### Astrocyte (GFAP, S100B) and neuronal (NfL) damage markers

In the acute stage, GFAP levels were strongly increased in NMOSD versus MOGAD, MS and SC (*P*_all_ < 0.009). For S100B this was only the case for NMOSD versus MOGAD (*P* < 0.001), with levels in the latter disease being below those of SC, while levels in NMOSD versus MS and SC were similar (Fig. 2). In the subacute/chronic stage, GFAP levels were similarly high across the three diseases, being significantly increased for MS (*P* = 0.002) and NMOSD (*P* = 0.015) versus SC. Levels of S100B were lower in NMOSD versus MS (*P* = 0.008) and SC (*P* = 0.051), but similar versus MOGAD. In the acute stage, NfL levels were higher in NMOSD versus MOGAD (*P* = 0.004), trended higher versus MS and were increased in all three diseases versus SC (*P*_all_ < 0.005) (Fig. 2), as well as in the subacute/chronic stage (*P*_all_ < 0.007, MOGAD > MS > NMOSD); however, the differences among the three diseases were not significant.

#### Complement factors C5 and C5a

C5 and C5a levels were strongly correlated within SC and all disease groups (*rho* = 0.95–0.99; *P*_all_ < 0.001, Supplementary Fig. 2) and were similarly increased in acute stages of MOGAD and NMOSD, but not in MS, versus SC (Fig. 2). In the subacute/chronic stage, C5 (*P*_all_ ≤ 0.047) and C5a (*P*_all_ ≤ 0.023) had higher levels in all three diseases (NMOSD > MOGAD > MS) versus SC. Correlations of C5 and C5a with GAM, ADM and NfL in diseases are shown in Supplementary Table 3. In acute MOGAD, C5 and C5a levels correlated with those of GAM, except for MPO missing significance levels (*P*_C5_ = 0.097, *P*_C5a_ = 0.090), ADM and NfL; in the subacute/chronic stage there was no positive correlation. In NMOSD, C5 and C5a levels showed a sustained, almost stable increase over MS across the 60-day analysis time (see later), and hence were not correlated with those of other markers, both in acute and subacute/chronic stages, except for NGAL (*P*_C5/C5a_ < 0.002) in the acute stage. In MS, no coherent pattern of positive correlation of C5/C5a with other compounds was observed: only NGAL correlated in the acute stage with C5 and C5a (*P*_both_ < 0.001); in the subacute/chronic stage this was the case for MMP-8 (*P*_both_ < 0.001) and nEla (*P*_C5a_ = 0.045).

#### Temporal dynamics of biomarker levels in relation to time between disease exacerbation and lumbar puncture

**Figure 3:**
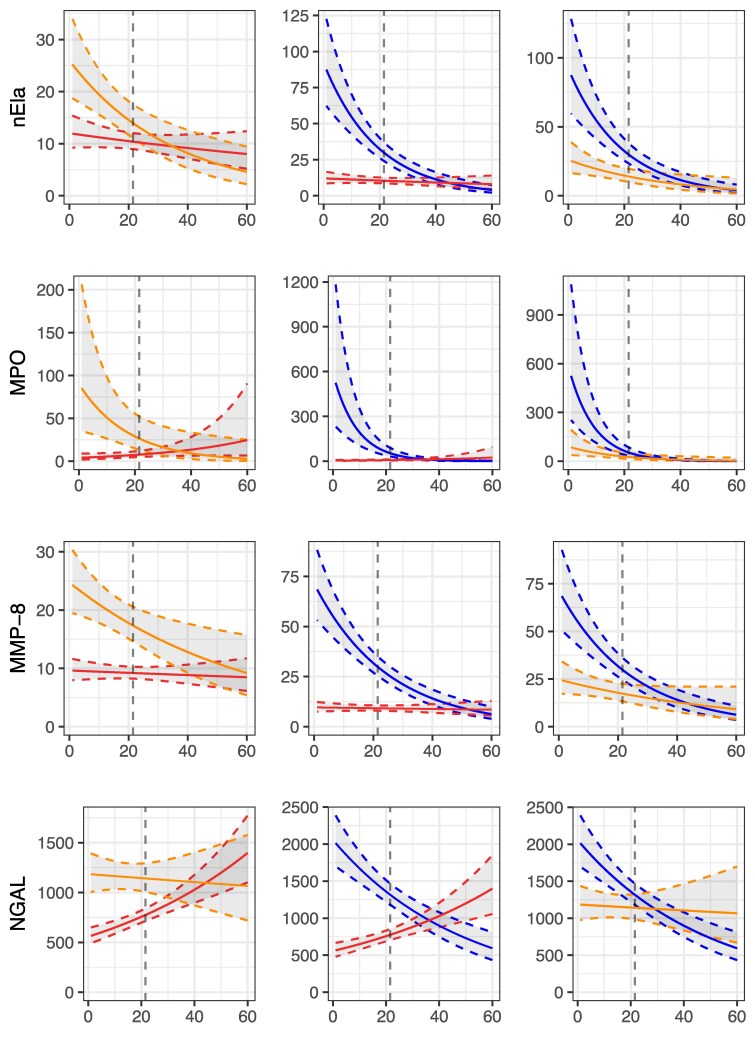
**Modelled kinetics of granulocyte activation markers (GAM) in CSF over 60 days following disease exacerbation**. Modelled CSF levels of neutrophil elastase (nEla), myeloperoxidase (MPO), matrix metalloproteinase-8 (MMP-8) and neutrophil gelatinase-associated lipocalin (NGAL) are shown for neuromyelitis optica spectrum disorder (NMOSD) (orange line), myelin oligodendrocyte glycoprotein antibody-associated disease (MOGAD) (blue line) and multiple sclerosis (MS) (red line). The *x*-axis represents days post-exacerbation (0–60 days), with the vertical dashed line marking the transition from the acute (≤21 days) to the subacute/chronic stage (>21 days). Biomarker values (*y*-axis) are plotted in pg/ml on a log-transformed scale. Solid lines represent modelled trajectories, with shaded areas denoting 95% confidence intervals. Distinct kinetic patterns are observed: Pattern 1 (nEla, MPO, MMP-8) shows early elevation in MOGAD and NMOSD but relative stability in MS; Pattern 2 (NGAL) shows early elevation in MOGAD and NMOSD with late-phase increase in MS.

**Figure 4:**
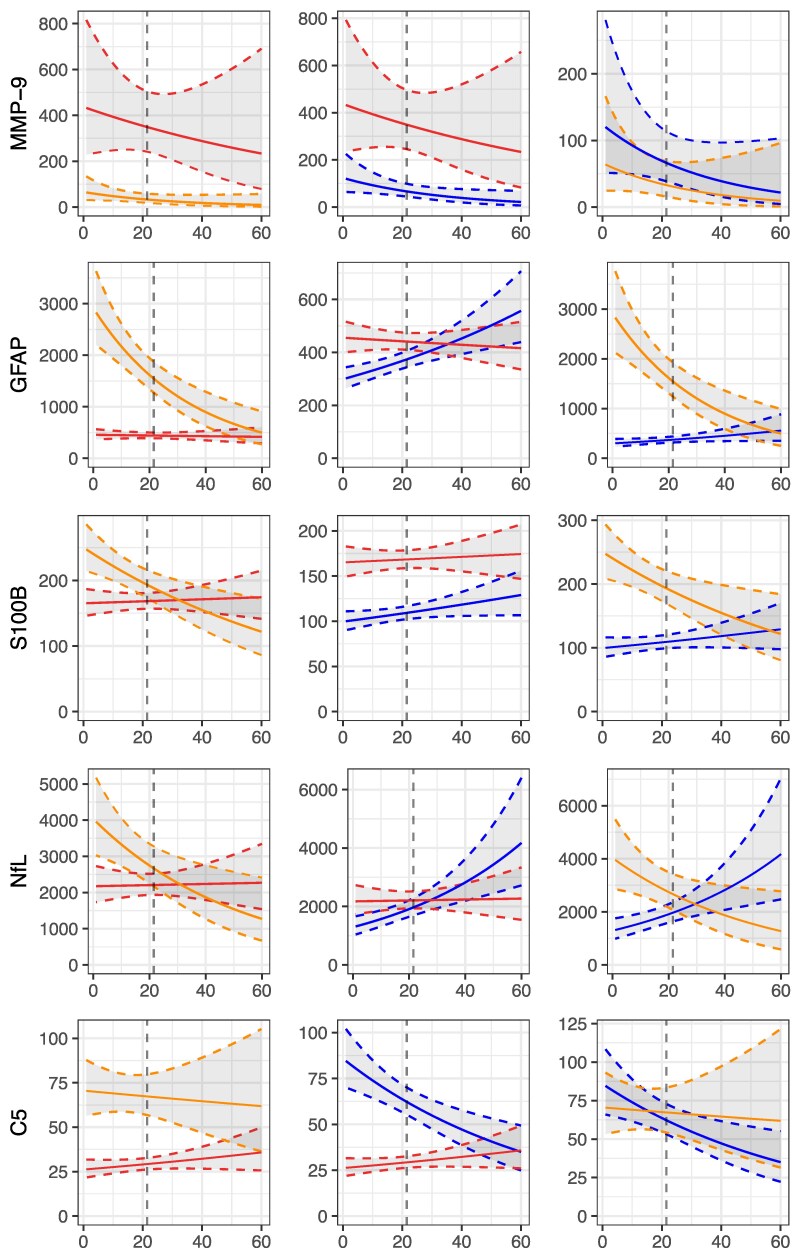
**Modelled kinetics of tissue damage markers and complement factor C5 in CSF over 60 days following disease exacerbation.** Modelled CSF levels of matrix metalloproteinase-9 (MMP-9), glial fibrillary acidic protein (GFAP), S100 calcium-binding protein B (S100B), neurofilament light chain (NfL) and complement C5 are shown for neuromyelitis optica spectrum disorder (NMOSD) (orange line), myelin oligodendrocyte glycoprotein antibody-associated disease (MOGAD) (blue line) and multiple sclerosis (MS) (red line). The *x*-axis represents days post-exacerbation (0–60 days), with the vertical dashed line indicating the transition from the acute (≤21 days) to the subacute/chronic stage (>22 days). Biomarker concentrations (*y*-axis) are plotted in pg/ml, except for C5, which is presented in ng/ml, using a log-transformed scale. Solid lines represent modelled trajectories, and shaded areas denote 95% confidence intervals. Distinct kinetic patterns are observed: MMP-9 remains chronically elevated in MS; GFAP and S100B show acute-stage increases predominantly in NMOSD; NfL is elevated across all diseases; and C5 increases during acute exacerbations in MOGAD and NMOSD relative to MS.

In NMOSD, GAM and many other biomarkers undergo rapid changes after disease exacerbation^12^; the categorical analysis (Figs 1 and 2) neglects these kinetic changes that lead to an underestimation of their capacity to differentiate the three diseases at stake. We therefore ran a time-dependent model applying a window of up to 60 days after disease exacerbation. In the acute stage GAM discriminated MOGAD and NMOSD from MS based on the non-overlapping pointwise 95% confidence intervals (CIs) within the acute disease stage; the same was the case for C5 and C5a (Figs 3 and 4). This pattern was characterized by peak levels of GAM except for MMP-9 at onset of disease exacerbation of MOGAD and NMOSD with rapidly decreasing levels within the 21-day window. The increase of ADM markers GFAP and S100B in NMOSD allowed to differentiate it versus MOGAD and MS (Fig. 4). While NGAL decreased in subacute/chronic stages of NMOSD and MOGAD, levels increased in MS over time. MMP-9 showed a third pattern with sustained elevated levels in MS over those in NMOSD and MOGAD throughout the entire 60-day period. NfL levels overlapped across the three diseases and therefore did not allow for a diagnostic differentiation.

### Capacity of biomarkers to differentiate acute stages of MOGAD, NMOSD and MS

We next explored the diagnostic value of single marker concentrations and of their composites to differentiate MOGAD and NMOSD from MS, expressed as ROC curves, respective AUC, and sensitivity and specificity (Supplementary Table 5). To simulate a situation of unclear differential diagnosis at first disease exacerbation, we restricted the analyses to patients in acute disease. While individual markers showed only moderate capacity to discriminate between disease groups, the combination of GAM (composite 1) provided good to excellent discrimination between MOGAD/NMOSD versus MS (AUC = 0.874), MOGAD versus MS (AUC = 0.923) and NMOSD versus MS (AUC = 0.825); the addition of C5 to composite 1 (composite 2) led to a further increase of AUC values (AUC_MOGAD/NMOSDversusMS_ = 0.880; AUC_MOGADversusMS_ = 0.925; AUC_NMOSDversusMS_ = 0.837).

The composite of GFAP and S100B (composite 3) showed AUC values of 0.897 for the differentiation of NMOSD versus MS, and 0.843 for NMOSD versus MOGAD.

We then evaluated the utility of these algorithms to differentiate MOGAD and NMOSD patients as ‘not MS’ using the Youden index of AUCs as cut-off. When MOGAD and NMOSD were compared jointly versus MS, 83.7% and 88.7% were identified as being ‘not MS’ by composite 1 and 2, respectively. When MOGAD and NMOSD were compared separately versus MS, 92.5% (in both composites) of MOGAD, and 74.2% and 77.4% of NMOSD were identified as ‘not MS’ by these algorithms. Accordingly, 62.5% (NMOSD versus MS) to 75.0% (MOGAD/NMOSD versus MS) of aAQP4^−^ NMOSD patients were identified as ‘not MS’ by composite 2, and 50.0% and 62.5% by composite 3 as ‘not MS’ and ‘not MOGAD’, respectively.

### Association between biomarker levels and disability status

**Figure 5:**
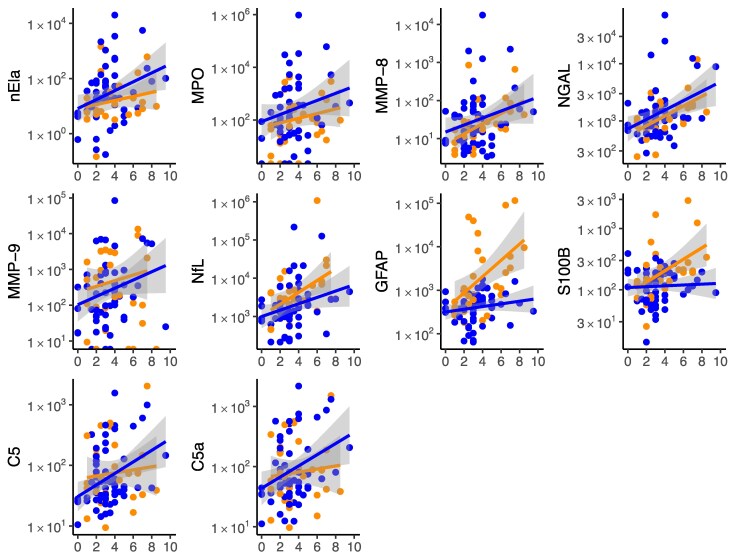
**Associations between CSF biomarker levels and disability scores (EDSS) in myelin oligodendrocyte glycoprotein antibody-associated disease (MOGAD) and neuromyelitis optica spectrum disorder (NMOSD)**. Scatter plots showing CSF biomarker concentrations plotted against Expanded Disability Status Scale (EDSS) scores at the time of lumbar puncture in patients with MOGAD (blue points and regression lines) and NMOSD (orange points and regression lines). Solid lines represent linear regression fits with shaded areas denoting 95% confidence intervals. Biomarkers shown include neutrophil elastase (nEla), myeloperoxidase (MPO), matrix metalloproteinase-8 (MMP-8), neutrophil gelatinase-associated lipocalin (NGAL), matrix metalloproteinase-9 (MMP-9), neurofilament light chain (NfL), glial fibrillary acidic protein (GFAP), S100 calcium-binding protein B (S100B), complement factor C5 and complement activation product C5a. Biomarker concentrations (*y*-axis) are presented in pg/ml, except for C5 (ng/ml). See Supplementary Table 4 for Spearman correlation coefficients and corresponding *P*-values.

We restricted the analysis of association of biomarker levels with EDSS scores at LP to patients with ≤5 years of disease duration due to the known lower rate of EDSS increase over time in all three diseases.^22,23^ The correlation of levels of markers with effector capacity for tissue damage (nEla, MPO, MMP-8, NGAL, MMP-9), tissue damage markers (NfL, GFAP, S100B) and C5/C5a with EDSS scores at LP is shown for the overall MOGAD and NMOSD patient groups in Fig. 5 and Supplementary Table 6. In the joint analysis of MOGAD and NMOSD patients, levels of GAM [*rho* = 0.24–0.39, *P*_all_ ≤ 0.024, except for MMP-9 (*rho* = 0.21, *P* = 0.051)], were correlated with the EDSS score. This was also the case for C5 (*rho* = 0.24, *P* = 0.024), while for C5a there was a trend (*rho* = 0.19, *P* = 0.073). When the analysis was limited to acute disease stage patients, NfL (*rho* = 0.35, *P* < 0.01), C5 (*rho* = 0.32, *P* = 0.020), MMP-8 (*rho* = 0.32, *P* = 0.021), MMP-9 (*rho* = 0.28, *P* = 0.041) and NGAL (*rho* = 0.46, *P* < 0.01), but not nEla (*P* = 0.376) and MPO (*P* = 0.085), correlated with EDSS scores.

The increased release of ADM in NMOSD, but not in MOGAD (Figs 2 and 4) finds its correlate in the discordant pattern for their association with the EDSS scores: in NMOSD ADM levels correlated with actual disability in all patients (*rho*_GFAP, S110B_ = 0.57, *P*_both_ < 0.01) and partly in acute patients (GFAP *rho* = 0.41, *P* = 0.089, S100B *rho* = 0.49, *P* = 0.039), while this was not the case for MOGAD (Fig. 5 and Supplementary Table 6B).

In contrast, in the overall MS group (*n* = 91) the EDSS was not correlated with biomarker levels, except for MMP-8 (*rho* = 0.30, *P* ≤ 0.01), and for GFAP (*rho* = 0.24, *P* = 0.022). Similarly, in acute stage MS (*n* = 32) only MMP-9 (*rho* = 0.60, *P* < 0.01) correlated with the EDSS, while there was a trend for MMP-8 (*rho* = 0.35, *P* = 0.052) and NfL (*rho* = 0.32, *P* = 0.072).

### Chronic immunomodulatory therapy prior to lumbar puncture and aAQP4-serostatus as potential confounding factors

Biomarker levels were not different in MOGAD and NMOSD patients with versus without chronic therapy prior to LP, both in acute and subacute/chronic phases (Supplementary Fig. 3). Accordingly, the aAQP4-serostatus in NMOSD was not associated with different biomarker levels except for nEla and MPO in the acute stage (Supplementary Table 4), likely due to sampling bias (Supplementary Fig. 1).

## Discussion

**Figure 6:**
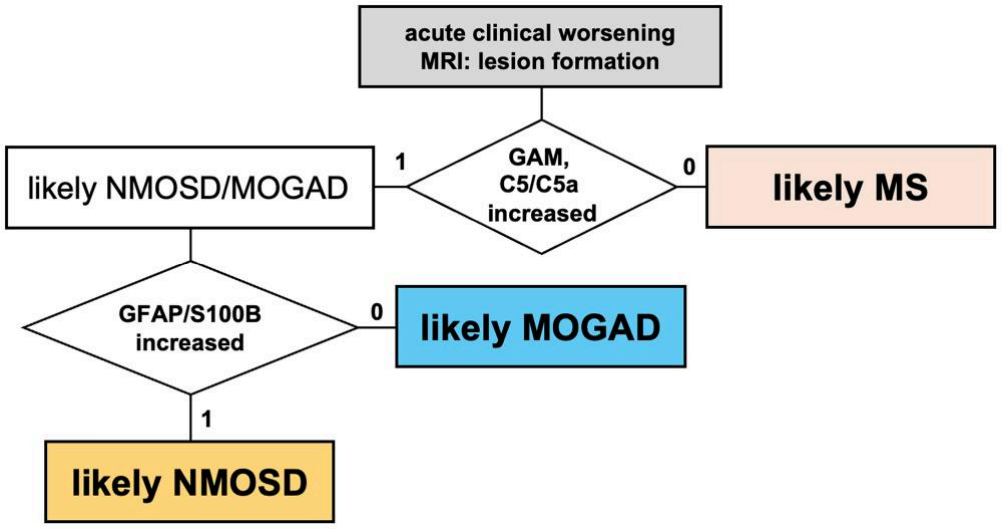
**Proposed diagnostic flow chart for differentiating MOGAD, NMOSD and MS based on CSF biomarkers.** Flow diagram illustrating a proposed decision pathway for the differential diagnosis of myelin oligodendrocyte glycoprotein antibody-associated disease (MOGAD), neuromyelitis optica spectrum disorder (NMOSD) and multiple sclerosis (MS) in patients presenting with acute clinical worsening and MRI evidence of new lesion formation. Initial separation is based on CSF levels of granulocyte activation markers (GAM) and complement factors C5/C5a. If elevated, differentiation between MOGAD and NMOSD is guided by astrocytic damage markers GFAP and S100B. Absence of biomarker increases suggests a diagnosis of MS.

Our findings demonstrate that a composite of GAM and complement factor C5 levels in CSF reliably distinguishes acute MOGAD from MS, and confirm previous results in an independent cohort that they have similar utility in distinguishing NMOSD from MS.^12^ ADM form a second tier of markers, that allow separating NMOSD from MOGAD and MS. Importantly, this diagnostic pattern also applies to the majority of NMOSD patients who test negative for aAQP4. This supports the sequential use of these markers in a diagnostic flow chart to differentiate the three diseases in acute stage presentations (Fig. 6). Moreover, GAM and C5/C5a concentrations are associated with the current level of disability in MOGAD and NMOSD patients, but not in MS.

Expression levels of individual biomarkers are often incongruent within a given patient, as shown both in the present dataset and in our earlier heat map analysis.^24^ Instead, composites of GAM and C5, and of ADM provide higher accuracy to differentiate between acute disease stages compared with single markers, as they compensate for two confounding factors: (i) LPs were performed at different time points of disease evolution following symptom onset; and (ii) the decline of individual markers follows divergent kinetics, with short apparent half-lives for markers such as MPO and S100B, and longer durations for markers like GFAP and NfL.^25,26^ These composite markers provide equal or higher sensitivity than most aAQP4 and aMOG tests, including live-cell assays,^27,28^ although specificity is slightly lower. We chose not to include time since symptom onset in the current ROC analyses, as doing so typically improves specificity at the group level,^12^ but is of limited utility for individual-level clinical decision-making. Importantly, this biomarker approach offers two main advantages over autoantibody-based diagnostics. First, it enables the differentiation versus MS in the 21%–40% of NMOSD patients who test negative for aAQP4.^29,30^ Second, and critically, it can deliver a differential diagnosis at the time of clinical presentation, on the day of symptom onset, using an assay platform that is rapid, robust and easy to operate, where timely initiation of therapy is crucial to contain the degree of disability in MOGAD and NMOSD.^4,5,7^ This stands in contrast to cell-based antibody assays, which require specialized laboratory infrastructure, trained personnel and often have prolonged turnaround times.^31,32^

MMP-9 is chronically upregulated in acute and stable MS^33^ a finding consistent with the present results where MMP-9 levels were always higher in MS versus MOGAD and NMOSD, suggesting that cellular sources other than granulocytes are quantitatively more relevant. Accordingly, levels of NGAL were higher in acute stages of MOGAD and NMOSD versus MS, indicative of a granulocytic source while its increase in the subacute/chronic stage of MS may be due to other neural cells contributing to neurodegeneration via myelination-dependent pathways.^34^ Surprisingly, we observed slightly higher levels of GAM in MOGAD than in NMOSD, despite the clinical observation that exacerbations in NMOSD are typically more severe.^35^ Our initial hypothesis that higher pre-treatment rates in NMOSD might reduce GAM and C5/C5a levels was not supported, as biomarker levels showed wide overlap between treated and untreated patients. An alternative explanation could be the age-related decline in immune activation, given that NMOSD patients were significantly older than those with MOGAD.

Autoantibody titres do not correlate with actual disease severity or disease course in either NMOSD or MOGAD.^29,36^ In contrast, the correlation of GAM levels with the disability scores at the time of LP in MOGAD and NMOSD reinforces the concept of their role as effectors of tissue damage. Granulocytes are the earliest infiltrating cells into NMOSD-like brain lesions in murine models;^15^ systemic granulocyte depletion preserved blood–brain barrier integrity and reduces lesional damage, while induction of a neutrophilic state led to increased neural damage.^14^ Complement-mediated spinal cord damage in an *ex vivo* model of NMOSD was potentiated by the addition of granulocytes or nEla.^16^ In all these models tissue damage could be partially prevented by inhibitors of nEla (Sivelestat) or cathepsin G.^14-16^ Sivelestat has also been shown to be efficacious in an *in vivo* model of NMOSD, but not in MS-like experimental autoimmune encephalomyelitis.^13^

It has been proposed that aAQP4^−^ NMOSD represents a distinct entity from aAQP4^+^ NMOSD, based on lower GFAP levels in CSF and serum, and differences in other neurodegeneration-related markers.^37,38^ However, these findings have not been consistently replicated,^39^ including in our current dataset. While we cannot determine whether serostatus defines distinct pathological subtypes, the consistent upregulation of GAM and complement factors C5 and C5a across groups supports a shared inflammatory profile of signalling and effector compounds associated with disease severity.

Complement factor C5 activation products C5a_Arg_ and C5a_des-Arg_ are potent endogenous degranulation and chemotactic agents for granulocytes.^13,40,41^ Others have found that C5a CSF levels are equally increased in acute MOGAD and NMOSD, but not in MS,^42,43^ findings that were confirmed here. However, our modelling revealed distinct kinetics between the diseases: C5/C5a levels in MOGAD increased transiently, mirroring GAM kinetics, while their elevation in NMOSD was sustained, explaining their correlation with GAM only in MOGAD. The association of C5/C5a levels with the degree of concurrent disability in MOGAD and NMOSD in the present results and by others^42^ may also have implications on the choice of acute therapy. C5-activation inhibitors have shown striking efficacy in acute NMOSD, particularly in refractory cases,^44-46^ and they are now recommended as a more efficacious, biology-targeting approach to contain acute disease activity and long-term disability in NMOSD.^47^ This strategy has now also been proposed for MOGAD patients experiencing severe exacerbations.^7^

An additional finding of our study is that nEla was slightly but significantly elevated in MS compared with SC, suggesting a role for granulocytes as well in MS pathogenesis, particularly in later disease stages, where C5/C5a levels were also increased. This is in line with the correlation of CSF C5/C5a levels with longitudinal brain atrophy, contrast-enhancing lesion formation, paramagnetic rim lesions and secondary progressive MS,^48^ and the observation of leptomeningeal granulocyte infiltration in progressive MS that goes along with a higher degree of subpial cortical demyelination.^49^

### Limitations

This was an exploratory analysis, and we did not adjust for multiple comparisons. While the current results are fully congruent with those of an earlier, independent cohort,^12^ they should be interpreted with caution. The diagnostic capacity of GAM was not evaluated in seronegative MOGAD, i.e. in 11%–28% of patients where aMOG expression is restricted to CSF.^50-53^ We are currently extending our analyses to this subgroup to test the hypothesis that their GAM, MMP-9 and C5/C5a expression is comparable or higher than in aMOG-seropositive MOGAD. If confirmed, this would suggest that autoantibody detection in serum is unrelated to levels of these biomarkers and disease severity, like in NMOSD. For the transition into clinical practice, ROC-based cut-offs will have to be replaced by normative reference values, allowing for standardized interpretation in suspected MOGAD and NMOSD cases.

## Conclusions

Current findings establish the combination of GAM and C5 as alternative biomarkers for the differential diagnosis of acute-stage MOGAD and NMOSD versus MS, while elevated ADM levels differentiate NMOSD from MOGAD, independent of aAQP4 serostatus. Our findings suggest that C5/C5a measurements in CSF should be considered in view of biology-based therapeutic decision-making,^54^ as they reinforce the rationale for acute stage use of complement C5-activation inhibitors in severe presentations of MOGAD and NMOSD.

## Supplementary Material

awaf345_Supplementary_Data

## Data Availability

The datasets that support the findings of the current study are available from the corresponding author on reasonable request. The data are not publicly available because they contain information that could compromise the privacy of the study participants.
