# Supplementary material for: Granulocyte and astrocyte markers distinguish MOG-antibody disease and neuromyelitis optica from multiple sclerosis
Source: Brain. 2025 Sep 24;149(4):1319–31. doi: 10.1093/brain/awaf345 (PMC13058455; doi:10.1093/brain/awaf345)
Supplement: awaf345_Supplementary_Data [file awaf345_supplementary_data.pdf]

Supplementary Material

Granulocyte and astrocyte markers-distinguish MOG-antibody disease and neuromyelitis optica from MS

Roberto Furlan,[1] Sabine Schaedelin,[2] Jette Lautrup Frederiksen,[3] Mitsuru Watanabe,[4] Noriko Isobe,[4] Fredrik Piehl,[5] Katharina Fink,[5] Ellen Iacobaeus,[5] Björn Evertsson,[5] Mohsen Khademi,[5] Matteo Gastaldi,[6] Giacomo Greco,[6] Sara Mariotto,[7] Sara Carta,[7] Alessia Di Sapio,[8] Cecilia Irene Bava,[8] Lucia Giorgi,[8] Pascal Benkert,[2] Aleksandra Maleska Maceski,[9] Johanna Oechtering,[9] Eline Willemse,[9] Anne-Katrin Pröbstel,[10] Roxanne Pretzsch,[9] Annamaria Finardi,[1] Alessandra Mandelli,[1] Daniel C Anthony,[11] Jens Kuhle,[9] David Leppert[9]

Affiliations

1 Clinical Neuroimmunology Unit, Institute of Experimental Neurology, Division of Neuroscience, IRCCS Ospedale San Raffaele, and Vita e Salute San Raffaele University, 20132 Milan, Italy
2 Department of Clinical Research, University Hospital Basel, University of Basel, 4031 Basel, Switzerland
3 Department of Neurology at Rigshospitalet and Institute of Clinical Medicine, University of Copenhagen, 2100 Copenhagen, Denmark
4 Department of Neurology, Neurological Institute, Graduate School of Medical Sciences, Kyushu University, 812-8582 Fukuoka, Japan
5 Department of Clinical Neuroscience, Karolinska Institutet and Department of Neurology, Karolinska University Hospital, SE-171 76 Stockholm, Sweden
6 Multiple Sclerosis Center, IRCCS Mondino Foundation, and Department of Brain and Behavioural Sciences, University of Pavia, 27100 Pavia, Italy
7 Neurology Unit, Department of Neurosciences, Biomedicine and Movement Sciences, University of Verona, 37134 Verona, Italy
8 Department of Neurology, Regional Referral MS Center and CRESM BioBank, San Luigi Gonzaga University Hospital, 10043 Orbassano, Italy
9 Multiple Sclerosis Centre and Research Center for Clinical Neuroimmunology and Neuroscience (RC2NB), Departments of Biomedicine and Clinical Research, and Department of Neurology, University Hospital and University of Basel, 4031 Basel, Switzerland
10 Center of Neurology, Department of Neuroimmunology, University Hospital and University Bonn, 53127 Bonn, Germany
11 Department of Pharmacology, University of Oxford, OX1 3QT Oxford, United Kingdom

Index Page

Supplementary Results

Impact of immunomodulatory and corticosteroid therapy prior lumbar puncture on GAM levels 3

Comparison of aAQP4$^+$ vs aAQP4$^-$ NMOSD patients 3

Supplementary Tables

Supplementary Table 1 4
Demographic and clinical data on 'symptomatic controls'

Supplementary Table 2 5
Statistical analysis of MOGAD, NMOSD, MS and SC in acute (A) and s/c (B) stages

Supplementary Table 3 7
Correlation of C5 (A) and C5a (B) with other biomarkers by diagnosis in acute and overall or s/c stages

Supplementary Table 4 9
Levels of markers according aAQP4 serostatus

Supplementary Table 5 10
ROC analyses in acute disease phases to differentiate MOGAD and NMOSD from MS by GAM, and
to differentiate NMOSD from MS and MOGAD by astrocyte damage markers

Supplementary Table 6 11
Correlation of EDSS scores with biomarker levels

Supplementary Figures

Supplementary Figure 1 12
Time between disease exacerbation and lumbar puncture

Supplementary Figure 2 13
Correlation of C5 and C5a by diagnosis

Supplementary Figure 3 14
Biomarker levels categorised for disease stage and treatment in MOGAD, NMOSD and MS

Supplementary Figure 4 15
Heatmaps of expression levels of biomarkers in individual samples

Reference 16

Supplementary Results

Impact of immunomodulatory and corticosteroid therapy prior lumbar puncture on GAM levels

GAM levels both in acute and s/c stages showed a wide range that overlapped between those with vs without corticosteroid or immunomodulatory therapy prior LP among all three diseases (Supplementary Fig. 3). Similarly, this was the case for GFAP, S100B, and C5/C5a levels. None of the comparisons of biomarker levels between treated vs non-treated MOGAD and NMOSD patients were significant, or showed a trend supporting the hypothesis that chronic treatment decreased expression levels in acute or s/c phase. For NMOSD, these findings are congruent with those of our earlier study.[1] We therefore conclude that the higher GAM levels in MOGAD vs NMOSD (Fig. 1A) do not result from the higher percentage of NMOSD under pharmacological therapy.

Comparison of $aAQP4^+$ vs $aAQP4^-$ NMOSD patients

$aAQP4^-$ patients in acute stage (n=8) had statistically lower levels of nEla and MPO than those being $aAQP4^+$ (n=23), while for all other markers they were not different (Supplementary Table 4). The former results need to be interpreted with caution due to the low sample numbers available for this comparison. Further, in the earliest period after disease exacerbation, i.e. when levels of GAM are highest (Fig. 3), $aAQP^-$ samples were underrepresented (between day 0 to 3 after exacerbation all six samples originated from $aAQP4^+$ patients; at and below the 2nd quartile border (day 0 to 4.5 after exacerbation), only 1 out of 8 samples was from an $aAQP^-$ patient (Supplementary Fig. 1). In s/c stage there was no difference in function of the aAQP4 serostatus for any marker (not shown).

Supplementary Table 1 Demographic and clinical data on 'symptomatic controls'

SC#	Age (years); sex	Symptoms	Final diagnosis	Relevant co-medication	Interval between symptom onset and LP (days)
1	32, F	dizziness	no neurological disorder	no	637
2	20, F	paraesthesia right body side	no neurological disorder	no	2
3	20, F	subjective vision loss	transient psychogenic vision loss	no	51
4	23, F	pain and walking impairment	acute low back pain, no neurological disease	no	16
5	54, F	diplopia	no neurological disease	no	13
6	32, F	dizziness	dizziness in migraine with aura	no	13
7	48, F	pain and walking impairment	fibromyalgia	no	n/a
8	21, F	asthenia	chronic fatigue syndrome	no	63
9	49, F	pain and walking impairment	somatic symptom disorder	no	n/av
10	24, F	right hemiparesis	somatic symptom disorder and panic disorder	no	n/av
11	29, M	intermittent vision decrease	drusen	no	14
12	36, M	blurred vision, temporal headache	no neurological disorder	no	15
13	36, F	focusing problem, headache	no neurological disorder	no	48
14	25, F	twitch upper eyelids, eye pain	no neurological disorder	no	35
15	31, F	moving spots in visual field	no neurological disorder	no	35
16	34, M	monosymptomatic blurred vision	no neurological disorder	no	37
17	26, F	pulse headache, periorbital pain	no neurological disorder	no	28
18	35, F	confluent vision, focus problem	anisometry	no	25
19	81, M	slowly fading colour vision	staphyloma	no	240

Abbreviation: n/av = not available

Supplementary Table 2 Statistical analysis of MOGAD, NMOSD, MS and SC in acute (A) and s/c (B) stages

A:

N	MOGAD 40	NMOSD 31	MS 45	SC[a] 19	MOGAD vs MS	NMOSD vs MS	MOGAD vs NMOSD	MOGAD vs SC	NMOSD vs SC	MS vs SC
	median [IQR]				*P*-value					
nEla	52.2 [18.8, 162.0]	19.5 [7.5, 48.8]	19.5 [6.2, 19.5]	3.9 [2.8, 5.8]	<0.001	0.153	0.034	<0.001	<0.001	<0.001
MPO	249.0 [59.3, 1541.0]	113.0 [32.1, 380.5]	22.1 [0.5, 215.5]	23.3 [2.7, 38.0]	<0.001	0.018	0.051	<0.001	0.002	0.651
MMP-8	42.4 [15.2, 83.1]	18.9 [11.1, 39.3]	8.7 [6.0, 14.7]	7.2 [4.2, 13.0]	<0.001	0.001	0.035	<0.001	0.008	0.246
NGAL	1360.0 [1062.0, 2072.8]	1217.0 [814.5, 1699.5]	617.0 [497.0, 912.0]	771.0 [629.0, 956.5]	<0.001	<0.001	0.348	0.001	0.004	0.078
MMP-9	114.5 [33.8, 828.5]	115.0 [18.6, 1815.0]	482.0 [233.0, 1716.0]	45.6 [14.7, 225.0]	0.017	0.042	0.903	0.061	0.130	<0.001
GFAP	358.5 [193.8, 567.2]	1307.0 [573.0, 7765.5]	497.0 [374.0, 626.0]	350.0 [268.0, 412.5]	0.039	<0.001	<0.001	0.929	<0.001	0.009
S100B	105.4 [66.3, 160.2]	194.0 [139.5, 309.0]	172.0 [139.0, 208.0]	162.0 [130.0, 269.5]	<0.001	0.146	<0.001	0.001	0.424	0.763
NfL	1282.0 [730.5, 2813.0]	2815.0 [1869.5, 6900.0]	2016.0 [1260.0, 4465.0]	378.0 [317.5, 856.5]	0.044	0.097	0.004	0.005	<0.001	<0.001
C5	53.2 [32.0, 180.0]	52.1 [25.3, 96.3]	26.7 [16.8, 40.8]	20.8 [12.9, 40.0]	<0.001	0.001	0.391	<0.001	0.001	0.314
C5a	80.6 [36.3, 301.0]	48.1 [26.8, 105.4]	32.0 [18.2, 42.6]	24.8 [15.3, 49.4]	<0.001	0.005	0.112	<0.001	0.010	0.562

B:

N	MOGAD 31	NMOSD 17	MS 80	SC[a] 19	MOGAD vs MS	NMOSD vs MS	MOGAD vs NMOSD	MOGAD vs SC	NMOSD vs SC	MS vs SC
	median [IQR]				*P*-value					
nEla	19.5 [4.5, 29.6]	10.8 [5.1, 19.5]	19.5 [4.3, 19.5]	3.9 [2.8, 5.8]	0.642	0.537	0.572	0.005	0.017	<0.001
MPO	25.9 [2.0, 103.0]	15.4 [0.0, 53.7]	19.2 [2.4, 67.9]	23.3 [2.7, 38.0]	0.792	0.654	0.479	0.404	0.936	0.659
MMP-8	10.1 [7.1, 30.0]	12.3 [5.8, 28.3]	7.8 [5.1, 11.8]	7.2 [4.2, 13.0]	0.018	0.058	0.872	0.095	0.141	0.793
NGAL	849.0 [678.0, 1248.5]	810.0 [619.0, 1075.0]	883.5 [704.8, 1114.0]	771.0 [629.0, 956.5]	0.750	0.553	0.525	0.250	0.646	0.248
MMP-9	134.0 [17.6, 426.5]	191.0 [19.6, 878.0]	470.0 [144.2, 1078.5]	45.6 [14.7, 225.0]	0.005	0.160	0.510	0.161	0.109	<0.001
GFAP	511.0 [292.5, 831.5]	551.0 [382.0, 841.0]	539.0 [376.0, 698.2]	350.0 [268.0, 412.5]	0.631	0.474	0.438	0.080	0.015	0.002
S100B	152.0 [91.2, 185.0]	137.0 [71.1, 198.0]	179.5 [143.8, 232.8]	162.0 [130.0, 269.5]	0.008	0.031	0.674	0.062	0.051	0.986
NfL	1871.0 [1024.5, 5392.5]	1051.0 [504.0, 2607.0]	1443.0 [886.8, 2690.5]	378.0 [317.5, 856.5]	0.264	0.333	0.215	<0.001	0.007	<0.001
C5	39.7 [23.9, 61.9]	50.5 [31.9, 184.0]	31.2 [21.1, 46.9]	20.8 [12.9, 40.0]	0.160	0.012	0.267	0.016	0.004	0.047
C5a	46.6 [30.0, 97.5]	58.1 [33.5, 210.0]	42.0 [26.2, 68.1]	24.8 [15.3, 49.4]	0.127	0.097	0.889	0.007	0.007	0.023

All biomarker levels are expressed as median [IQR] in pg/ml except for C5 (ng/ml). [a]The SC group is identical in A and B.
Background colour of *P*-values related to positive association: green: ≤0.05; yellow: 0.05-0.10; red: >0.10.

Supplementary Table 3 Correlation of C5 (A) and C5a (B) with other biomarkers by diagnosis in acute and overall or s/c stages

A:

	MOGAD		NMOSD		MS	
	acute	s/c	acute	s/c	acute	s/c
N	40	31	31	17	45	78
nEla	0.32 [0.01, 0.58], 0.041	0.07 [-0.29, 0.41], 0.705	0.19 [-0.18, 0.51], 0.315	-0.25 [-0.65, 0.27], 0.342	0.21 [-0.09, 0.47], 0.170	0.19 [-0.04, 0.39], 0.102
MPO	0.27 [-0.05, 0.53], 0.097	0.11 [-0.25, 0.45], 0.550	-0.02 [-0.37, 0.33], 0.899	-0.13 [-0.58, 0.37], 0.614	-0.37 [-0.60, -0.08], 0.014	0.04 [-0.18, 0.26], 0.704
MMP-8	0.43 [0.13, 0.65], 0.006	0.17 [-0.19, 0.50], 0.349	0.28 [-0.09, 0.57], 0.132	0.45 [-0.04, 0.77], 0.070	0.16 [-0.14, 0.43], 0.305	0.47 [0.28, 0.63], <0.001
NGAL	0.52 [0.25, 0.72], <0.001	0.27 [-0.09, 0.57], 0.138	0.53 [0.21, 0.74], 0.002	0.29 [-0.22, 0.68], 0.252	0.56 [0.32, 0.73], <0.001	0.20 [-0.02, 0.40], 0.079
MMP-9	0.14 [-0.18, 0.43], 0.400	-0.01 [-0.37, 0.34], 0.947	0.16 [-0.20, 0.49], 0.377	0.22 [-0.29, 0.63], 0.403	0.17 [-0.13, 0.44], 0.257	0.08 [-0.15, 0.30], 0.487
GFAP	0.02 [-0.29, 0.33], 0.907	0.08 [-0.29, 0.42], 0.682	0.07 [-0.29, 0.41], 0.706	-0.01 [-0.48, 0.48], 0.984	0.20 [-0.10, 0.47], 0.190	0.04 [-0.19, 0.26], 0.744
S100B	-0.22 [-0.50, 0.10], 0.169	-0.34 [-0.62, 0.01], 0.060	-0.17 [-0.49, 0.20], 0.365	-0.41 [-0.74, 0.09], 0.102	0.22 [-0.08, 0.48], 0.154	-0.22 [-0.42, -0.00], 0.049
NfL	0.05 [-0.27, 0.36], 0.760	-0.06 [-0.40, 0.30], 0.756	0.12 [-0.25, 0.46], 0.541	0.40 [-0.10, 0.74], 0.112	0.12 [-0.18, 0.40], 0.416	0.12 [-0.10, 0.34], 0.278

B:

	MOGAD		NMOSD		MS	
	acute	s/c	acute	s/c	acute	s/c
N	40	31	31	17	45	78
nEla	0.35 [0.04, 0.59]<br>0.029	0.05 [-0.31, 0.39]<br>0.806	0.18 [-0.19, 0.50]<br>0.332	-0.24 [-0.64, 0.28]<br>0.362	0.14 [-0.16, 0.42]<br>0.359	0.23 [0.01, 0.43]<br>0.045
MPO	0.27 [-0.04, 0.54]<br>0.090	0.07 [-0.29, 0.41]<br>0.710	-0.02 [-0.37, 0.33]<br>0.900	-0.11 [-0.56, 0.39]<br>0.682	-0.39 [-0.62, -0.11]<br>0.009	0.01 [-0.22, 0.23],<br>0.995
MMP-8	0.44 [0.15, 0.66]<br>0.005	0.14 [-0.22,0.47]<br>0.444	0.29 [-0.07,0.58]<br>0.117	0.48 [-0.00,0.78]<br>0.053	0.12 [-0.18,0.40]<br>0.437	0.43 [0.23,0.59]<br><0.001
NGAL	0.52 [0.25, 0.72]<br>0.001	0.23 [-0.14, 0.54]<br>0.215	0.53 [0.22, 0.75]<br>0.002	0.32 [-0.19, 0.69]<br>0.215	0.55 [0.31, 0.73]<br><0.001	0.11 [-0.12, 0.32]<br>0.347
MMP-9	0.11 [-0.21, 0.40]<br>0.515	-0.06 [-0.41, 0.30]<br>0.748	0.16 [-0.20, 0.49]<br>0.385	0.21 [-0.30, 0.63]<br>0.412	0.14 [-0.16, 0.42]<br>0.342	0.10 [-0.13, 0.32]<br>0.383
GFAP	-0.06 [-0.36, 0.26]<br>0.716	0.01 [-0.35, 0.36]<br>0.970	0.05 [-0.31, 0.40]<br>0.786	0.04 [-0.45, 0.51]<br>0.884	0.22 [-0.08, 0.48]<br>0.142	-0.10 [-0.31, 0.13]<br>0.405
S100B	-0.27 [-0.54, 0.04]<br>0.086	-0.38 [-0.65, -0.03]<br>0.034	-0.19 [-0.51, 0.17]<br>0.296	-0.41 [-0.74, 0.09]<br>0.105	0.26 [-0.04, 0.51<br>0.088	-0.30 [-0.49, -0.08]<br>0.008
NfL	0.01 [-0.31, 0.33]<br>0.947	-0.09 [-0.43, 0.27]<br>0.618	0.13 [-0.25, 0.46]<br>0.509	0.43 [-0.07, 0.75]<br>0.087	0.15 [-0.15, 0.42]<br>0.338	0.03 [-0.20, 0.25]<br>0.814

Numbers are *rho* [CI] (first line) and *P*-values (second line) in each box. As shown in Supplementary Fig. 2 values of C5 and C5a are highly correlated and hence lead to the same pattern of correlation with referring markers. Background colour of *P*-values related to positive association: green: ≤0.05; yellow: 0.05-0.10; red: >0.10. Negative correlations are depicted in white background.

Supplementary Table 4 Levels of markers according aAQP4 serostatus

	acute		*P*-value
aAQP4 serostatus<br>*N*	-<br>8	+<br>23	
nEla	8.4 [5.3, 16.7]	19.5 [15.8, 88.8]	0.016
MPO	26.8 [5.4, 83.9]	168.0 [51.5, 512.5]	0.058
MMP-8	14.4 [9.7, 18.9]	20.2 [13.1, 65.2]	0.148
NGAL	857.0 [768.8, 1460.5]	1313.0 [925.0, 1867.0]	0.190
MMP-9	72.8 [0.0, 150.5]	192.0 [34.5, 2307.5]	0.220
GFAP	1017.5 [545.5, 1465.2]	1718.0 [663.5, 21945.0]	0.190
S100B	212.0 [125.5, 259.2]	194.0 [148.5, 498.0]	0.718
NfL	2094.0 [1449.0, 9063.0]	2860.0 [2032.0, 6656.0]	0.902
C5	37.8 [33.7, 57.5]	66.5 [24.4, 122.3]	0.588
C5a	41.2 [34.8, 57.8]	69.2 [24.5, 152.4]	0.619

Values represent median [IQR] in ng/ml for C5, and pg/ml for all other markers.
Background colour of *P*-values related to positive association: green: ≤0.05; yellow: 0.05-0.10; red: >0.10.

Supplementary Table 5 ROC analyses in acute disease phases to differentiate MOGAD and NMOSD from MS by GAM, and to differentiate NMOSD from MS and MOGAD by astrocyte damage markers

AUC [CI]		MOGAD/NMOSD vs MS	MOGAD vs MS	NMOSD vs MS	NMOSD vs MOGAD
nEla		0.676 [0.582, 0.770]	0.740 [0.628, 0.852]	0.592 [0.459, 0.726]	0.647 [0.518, 0.776]
MPO		0.721 [0.622, 0.819]	0.768 [0.666, 0.870]	0.661 [0.535, 0.786]	0.635 [0.506, 0.765]
MMP-8		0.780 [0.695, 0.865]	0.829 [0.732, 0.926]	0.718 [0.589, 0.846]	0.646 [0.515, 0.777]
NGAL		0.801 [0.716, 0.886]	0.810 [0.715, 0.905]	0.789 [0.685, 0.894]	0.565 [0.429, 0.701]
MMP-9		0.645 [0.544, 0.745]	0.650 [0.526, 0.774]	0.638 [0.498, 0.777]	0.492 [0.352, 0.631]
**Composite 1** (nEla+MPO+MMP-8+NGAL+MMP-9)<br>Sensitivity/Specificity<br>identified as 'not MS'<br>identified as 'NMOSD not MOGAD'<br>identified as NMOSD of aAQP4$^-$ patients	AUC [CI]<br>%<br>% (n/N)	0.874 [0.808, 0.940]<br>87.3/79.5<br>87.3 (62/71)<br>n/a<br>62.5 (5/8)	0.923 [0.867, 0.979]<br>92.5/81.8<br>92.5 (37/40)<br>n/a<br>n/a	0.825 [0.724 ,0.925]<br>74.2/86.4<br>74.2 (23/31)<br>n/a<br>50.0 (4/8)	0.690 [0.567, 0.814]<br>77.4/62.5<br>n/a<br>77.4 (24/31)<br>75.0 (6/8)
**Composite 2** (nEla+MPO+MMP-8+NGAL+MMP-9+C5)<br>Sensitivity/Specificity<br>identified as 'not MS'<br>identified as 'NMOSD, not MOGAD'<br>identified as NMOSD of all aAQP4$^-$ patients[a]	AUC [CI]<br>%<br>% (n/N)	0.880 [0.815, 0.945]<br>88.7/79.5<br>88.7 (63/71)<br>n/a<br>75.0 (6/8)	0.925 [0.870, 0.980]<br>92.5/84.1<br>92.5 (37/40)<br>n/a<br>n/a	0.837 [0.740, 0.935]<br>77.4/86.4<br>77.4 (24/31)<br>n/a<br>62.5 (5/8)	0.702 [0.581, 0.822]<br>77.4/57.5<br>n/a<br>77.4 (24/31)<br>87.5 (7/8)
GFAP<br>Sensitivity/Specificity<br>identified as 'not MS'<br>identified as 'not MOGAD'<br>identified as NMOSD of all aAQP4$^-$ patients[a]	AUC [CI]<br>%<br>% (n/N)		0.630 [0.505, 0.755]<br>40.0/91.1<br>40.0 (16/40)<br>n/a<br>n/a	0.796 [0.676, 0.916]<br>64.5/97.8<br>64.5 (20/31)<br>n/a<br>62.5 (5/8)	0.844 [0.753, 0.934]<br>71.0/87.5<br>n/a<br>71.0 (22/31)<br>62.5 (5/8)
S100B<br>Sensitivity/Specificity<br>identified as 'not MS'<br>identified as 'not MOGAD'<br>identified as NMOSD of all aAQP4$^-$ patients[a]	AUC [CI]<br>%<br>% (n/N)		0.750 [0.640, 0.859]<br>62.5/84.4<br>62.5 (25/40)<br>n/a<br>n/a	0.599 [0.459, 0.738]<br>25.8/100<br>25.8 (8/31)<br>n/a<br>1/8 (12.5%)	0.772 [0.661 ,0.884]<br>93.5/50.0<br>n/a<br>93.5 (29/31)<br>100 (8/8)
**Composite 3** (GFAP+S100B)<br>Sensitivity/Specificity<br>identified as 'not MS'<br>identified as 'not MOGAD'<br>identified as NMOSD of all aAQP4$^-$ patients[a]	AUC [CI]<br>%<br>% (n/N)		0.746 [0.635, 0.857]<br>62.5/84.4<br>62.5 (25/40)<br>n/a<br>n/a	0.897 [0.829, 0.966]<br>74.2/91.1<br>74.2 (23/31)<br>n/a<br>50.0 (4/8)	0.843 [0.751, 0.934]<br>71.0/87.5<br>n/a<br>71.0 (22/31)<br>62.5 (5/8)

Numbers are AUC [CI] values per box. AUC values ≥0.80 (very good to excellent performance) are with green background. Numbers of aAQP4$^-$ patients identified as NMOSD by algorithms are highlighted in blue.
ROC curves are calculated on parameters based on a logistic model. Youden-index as estimated is used as cut-off. [a]Patients were identified by AUC score values above cut-off, defined by the Youden-index. Abbreviation: n/a = not applicable.

Supplementary Table 6 Correlation of EDSS scores with biomarker levels

A: Compounds with tissue damaging capacity, NfL and complement factors C5/5a

	MOGAD+NMOSD			
	all phases *n*=88		acute phases *n*=53	
	rho	*P*-value	*rho*	*P*-value
nEla	0.24	0.024	0.12	0.376
MPO	0.29	<0.01	0.24	0.085
MMP-8	0.32	<0.01	0.32	0.021
NGAL	0.39	<0.01	0.46	<0.01
MMP-9	0.21	0.051	0.28	0.041
NfL	0.34	<0.01	0.35	<0.01
C5	0.24	0.024	0.32	0.020
C5a	0.19	0.073	0.25	0.074

B: Astrocyte damage markers

	NMOSD *n*=29		MOGAD *n*=59		NMOSD *n*=18		MOGAD *n*=35	
	all phases				acute phase			
	rho	*P*-value	*rho*	*P*-value	*rho*	*P*-value	*rho*	*P*-value
GFAP	0.57	<0.01	0.24	0.065	0.41	0.089	0.33	0.056
S100B	0.57	<0.01	0.09	0.483	0.49	0.039	0.17	0.333

Patients with disease duration of ≤5 years at LP were compared.
Background colour of *P*-values: green: ≤0.05: green; yellow: 0.05-0.10 ; red: >0.10.

Supplementary Figure 1 Time_between disease exacerbation and lumbar puncture

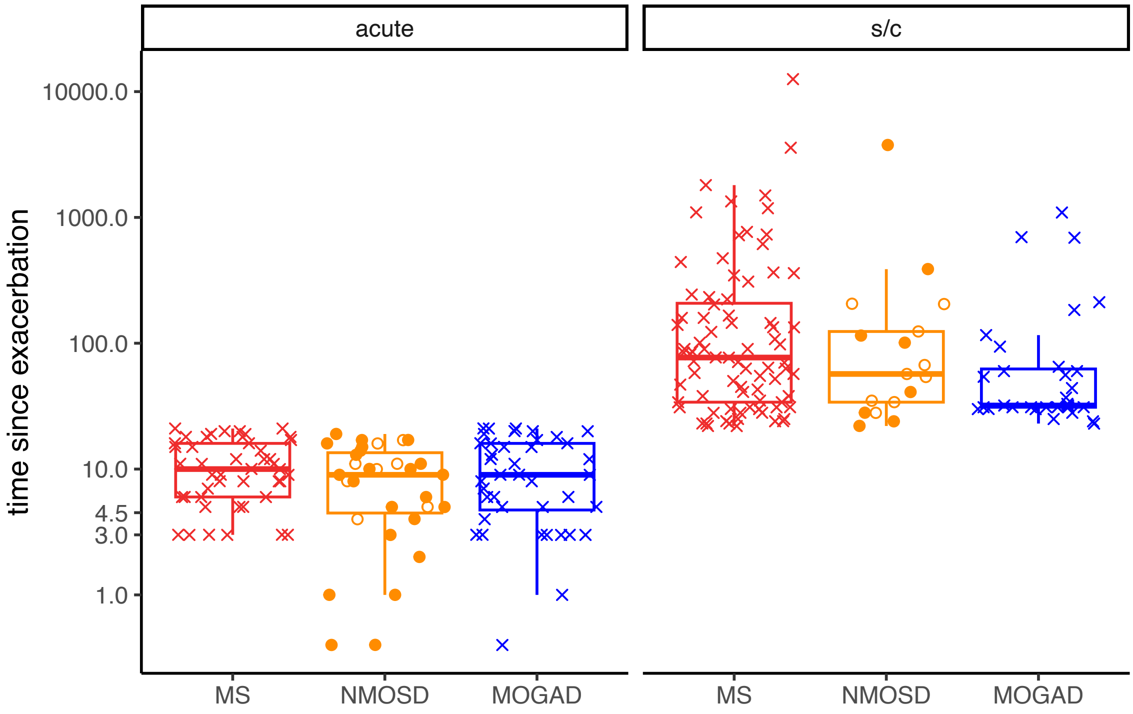

Distribution of intervals [days] between disease exacerbation and LP is similar across diseases for acute (left panel) and s/c (right panel) phase patients. For the acute phase group median intervals were 10 days for MS, and 9 days for NMOSD and MOGAD; the pairwise comparison of intervals was not significant (MS vs NMOSD: p=0.141, MS vs MOGAD: p=0.513, NMOSD vs MOGAD: p=0.457). For s/c patients median intervals were 77 days for MS, 57 days for NMOSD, and 32 days for MOGAD; the difference between MS and MOGAD was significant (p=0.021), but not for MS vs NMOSD (p=0.506) and NMOSD vs MOGAD (p=0.280).
Note that in acute stage NMOSD ≤1st quartile (0-4.5 days after disease exacerbation) 7 out of 8 patients were $aAQP4^{+}$ (●) ($aAQP4^{-}$: (○); in the time of 0-3 days post exacerbation all 6 samples originate from $aAQP4^{+}$ patients.

Supplementary Figure 2 Correlation of C5 and C5a by diagnosis

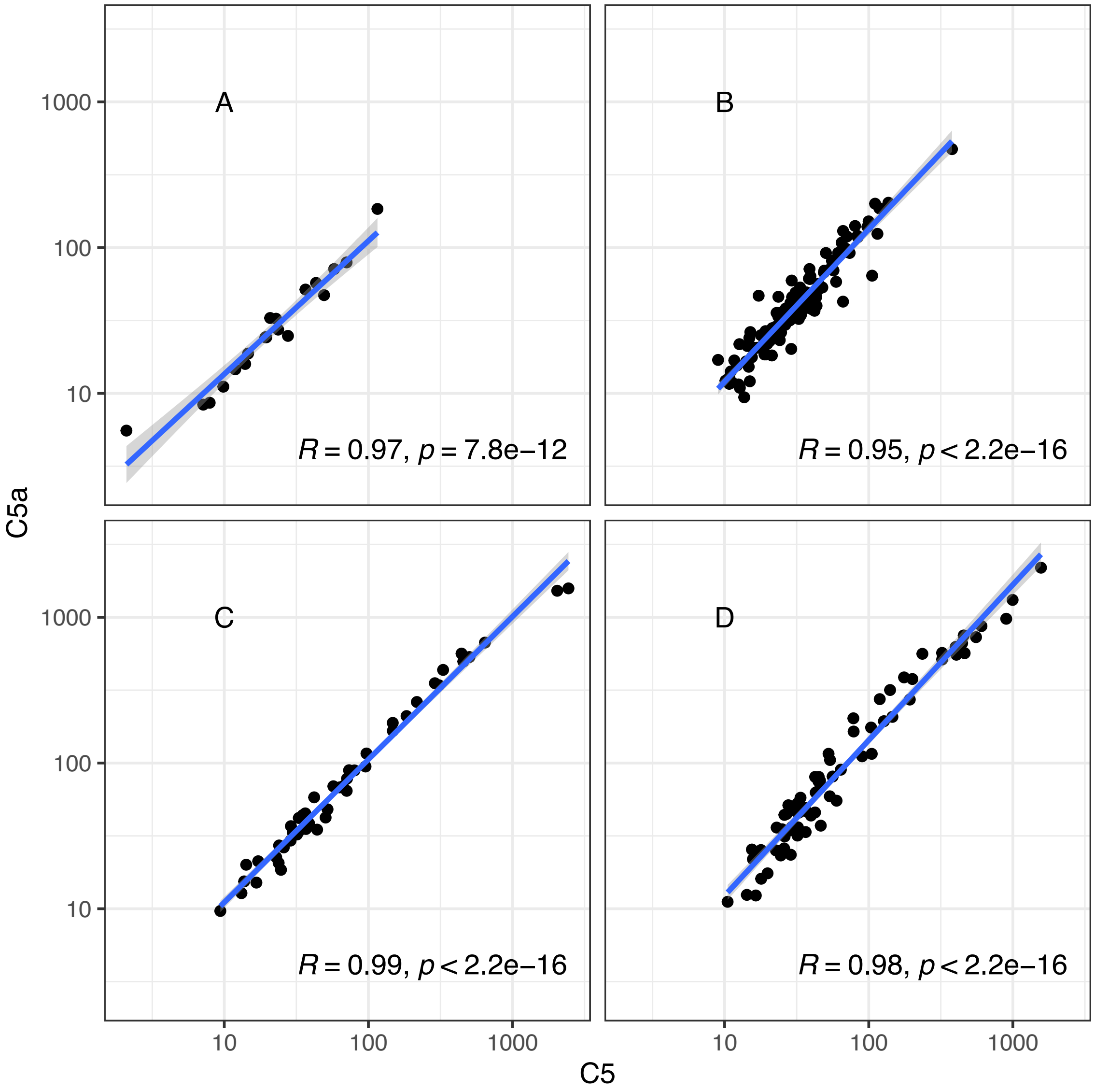

Correlation of C5a (y-axis) and C5 (x-axis) values in pg/ml and ng/ml, respectively, in (A) SC, (B) MS, (C) NMOSD, and (C) MOGAD.

Supplementary Figure 3 Biomarker levels categorised for disease stage and treatment in MS (●), NMOSD (●) and MOGAD (●)

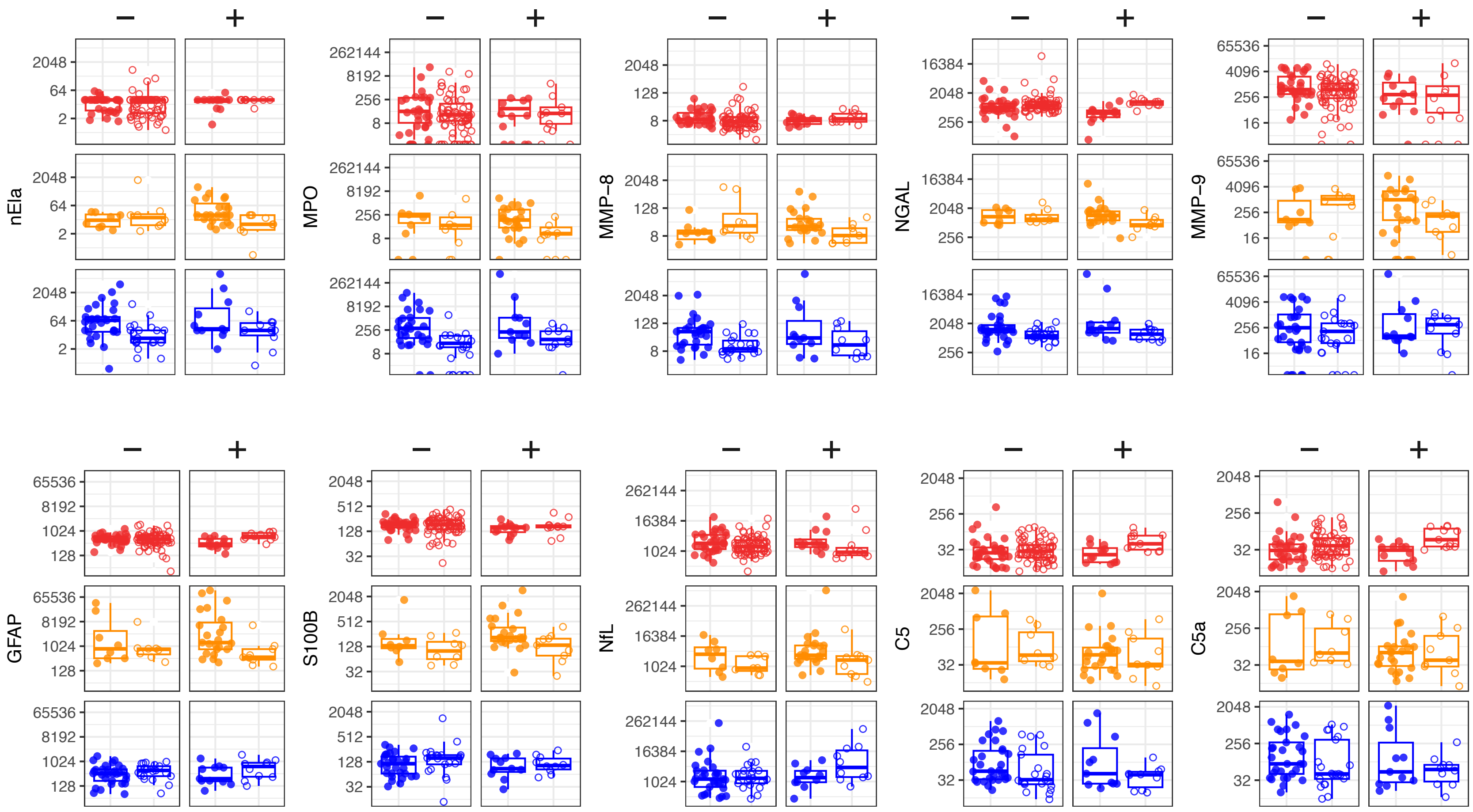

Values of biomarkers (acute stage: ● ; s/c phase: ○ on y-axis are in pg/ml, except for C5 that are in ng/ml. Column headers determine patients without (-) or with (+) immunomodulatory therapy at LP.

Supplementary Figure 4 Temporal heatmaps of expression levels of biomarkers in individual NMOSD and MOGAD CSF samples

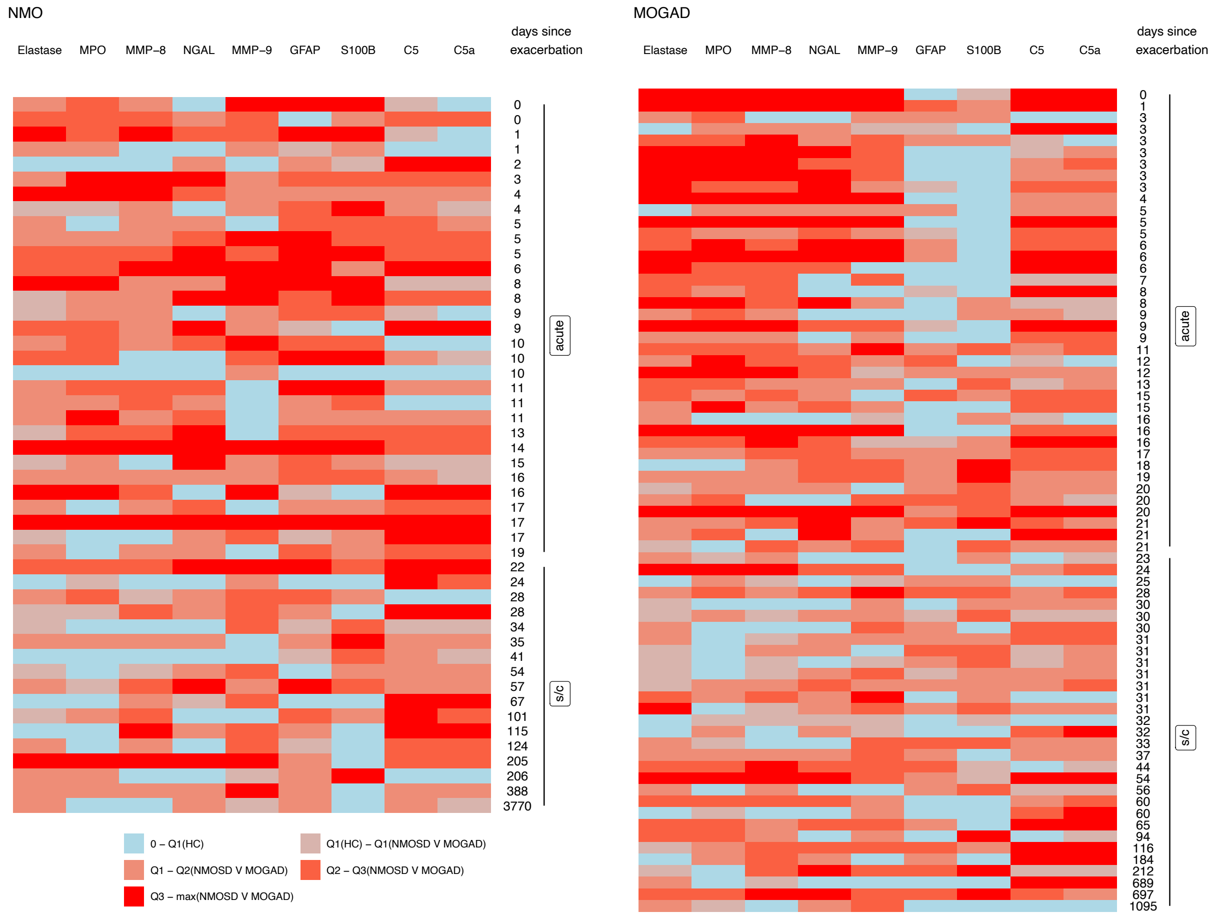

Heatmaps show biomarker levels of individual CSF samples from NMOSD (left) and MOGAD (right) patients, ordered by days between clinical exacerbation and lumbar puncture and by clinical stage (acute: ≤21 days; s/c: >21 days).
Note the low expression levels of GFAP and S100B in acute stages of MOGAD compared to NMOSD, while in function of time since disease exacerbation relatively higher levels occur in MOGAD.
Quartiles for NMOSD and MOGAD were defined for each marker individually by the respective higher value, i.e. Q*n* (NMOSD V MOGAD). Cut-off values for colour codes (all numbers are in pg/ml except for C5 which is in ng/ml):

Biomarker	Red (High)	Pink	Brown	Beige	Blue (Low)
nEla	>79.1	>19.5–79.1	>7.99–19.5	>3.5–7.99	0–3.5
MPO	>458	>95.4–458	>23.9–95.4	>9.2–23.9	0–9.2
MMP-8	>71.3	>24.1–71.3	>9.1–24.1	>6.0–9.1	0–6.0
NGAL	>1610	>1132–1610	>763.5–1132	>750.8–763.5	0–750.8
MMP-9	>1303	>145.5–1303	>24.7–145.5	>14-24.7	0–14
GFAP	>3703	>852.5–3703	>403.3–852.5	>319.5–403.3	0–319.5
S100B	>237.3	>183.5–237.3	>119.5–183.5	>105–119.5	0–105
C5 (ng/mL)	>147.6	>51.3–147.6	>29.0–51.3	>19.4–29.0	0–19.4
C5a (ng/mL)	>239.9	>62.7–239.9	>35.6–62.7	>24.4–35.6	0–24.4

Reference

1. Leppert D, Watanabe M, Schaedelin S*, et al*. Granulocyte activation markers in cerebrospinal fluid differentiate acute neuromyelitis spectrum disorder from multiple sclerosis. *J Neurol Neurosurg Psychiatry*. 2023; 94:726-737
